# Ginsenoside RG3 Synergizes With STING Agonist to Reverse Cisplatin Resistance in Gastric Cancer

**DOI:** 10.1002/fsn3.4744

**Published:** 2025-01-20

**Authors:** Zhongqi Lu, Yihang Fu, Qiang Fu, Ying Chang, Meihua Zhang, Tiefeng Jin

**Affiliations:** ^1^ Department of Central Laboratory Yanbian University Hospital Yanji China; ^2^ Key Laboratory of the Science and Technology Department of Jilin Province Yanji China; ^3^ Department of Ultrasound Medicine Shaanxi Provincial People's Hospital Xi'An Shaanxi China; ^4^ Department of Ultrasound Medicine Yanbian University Hospital Yanji Jilin China; ^5^ Department of Health Examination Centre Yanbian University Hospital Yanji China

**Keywords:** cisplatin resistance, gastric cancer, ginsenoside RG3, immunotherapy, STING agonist

## Abstract

This study investigates the synergistic inhibitory effects of combining the stimulator of interferon genes (STING) agonist cyclic diadenylate monophosphate (c‐di‐AMP) and ginsenoside RG3 on cisplatin (DDP)‐resistant gastric cancer (GC) cells. The objective is to identify novel therapeutic targets and offers insights for the clinical management of DDP resistance. Various techniques were employed, including western blot, MTT assay, colony formation assay, scratch assay, transwell assay, tubule formation assay, flow cytometry, Hoechst 33342 fluorescence staining, and in vivo experiments, to investigate the potential mechanisms and effects of the combined application of the STING agonist and ginsenoside RG3 in reversing cisplatin resistance in gastric cancer. The combination markedly suppressed key malignant behaviors, including proliferation, migration, invasion, and angiogenesis of SGC‐7901/DDP cells. Additionally, this treatment inhibited the epithelial‐mesenchymal transition (EMT) process and stem cell‐like characteristics of SGC‐7901/DDP cells, while downregulating the expression of resistance‐related proteins. The STING agonist effectively suppresses the growth and proliferation of gastric cancer cells. Ginsenoside RG3, well‐documented for its multifaceted properties, including antioxidant, anti‐aging, and anti‐cancer effects, is widely used in cancer treatment and in managing chemotherapy‐related side effects. Furthermore, RG3 enhances anti‐tumor immunity by regulating signal transduction. This study comprehensively evaluated the efficacy of the STING agonist and RG3 combination through in vitro and in vivo experiments, demonstrating significant inhibition of malignant progression and reversal of drug resistance in gastric cancer. These findings offer a robust theoretical foundation for clinical applications and highlight new therapeutic targets for future research.

## Graphical abstract

Our study demonstrates the synergistic potential of the STING agonist c‐di‐AMP in conjunction with the traditional Chinese medicine ginsenoside Rg3 to effectively reverse cisplatin resistance in SGC‐7901/DDP cells. This collaborative approach presents a novel and promising strategy for immunotherapy, particularly in addressing the clinical challenges associated with chemotherapy‐resistant gastric cancer. Further exploration and validation of this combination could pave the way for innovative and more effective therapeutic interventions in the field of gastric cancer treatment.

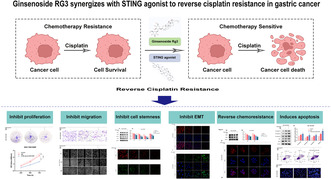

## 1 Introduction

### 1.1 Current Research Status of Gastric Cancer

Gastric cancer is a common malignant tumor of the digestive system, ranking fifth in global cancer incidence and the fourth most common cause of cancer‐related mortality (Sung et al. 2021). Studies highlight notable regional disparities in the incidence of gastric cancer, with over 69% of cases reported in East Asia and South‐Central Asia in 2020 (Yang, Zhao, et al. 2023). The onset of gastric cancer is often insidious, resulting in late‐stage diagnoses and unfavorable prognoses in the majority of cases (Guan, He, and Xu 2023). The primary treatment for gastric cancer is surgical resection. Additionally, systemic therapies, including chemotherapy, targeted therapy, and immunotherapy, have advanced considerably in recent years. However, postoperative metastasis or recurrence continues to pose a significant challenge in clinical practice. Immunotherapy plays a supportive role in chemotherapy by targeting residual non‐metastatic cancer cells and preventing the spread or relapse of cancer (Smyth et al. 2020; Zhao, Lv, and Lin 2020). Furthermore, drug resistance remains a critical barrier to achieving optimal therapeutic outcomes for gastric cancer (Zhang et al. 2024). It substantially compromises the efficacy of chemotherapy agents, often leading to tumor metastasis or recurrence in many patients (Zhang et al. 2021).

Cisplatin is a commonly used chemotherapeutic agent for gastric cancer, with its anti‐tumor effects mediated by multiple mechanisms. However, resistance to cisplatin often develops during chemotherapy due to factors such as reduced drug uptake, increased drug efflux, enhanced DNA damage repair, and alterations in apoptotic signaling pathways, presenting substantial challenges to effective clinical management (Fan et al. 2021). Research has underscored the pivotal role of microRNAs in mediating cisplatin resistance in gastric cancer patients (Liu, Li, and Tang 2023). Additionally, factors such as DNA methylation (Choi et al. 2017), E3 ubiquitin ligases (Wang et al. 2021), and the IL‐6/STAT3 axis (Laurino et al. 2023) have been closely implicated in cisplatin resistance in gastric cancer. Therefore, there is an urgent need to identify effective therapeutic targets to improve cisplatin sensitivity in gastric cancer.

### 1.2 Advancements in Research on STING Agonists

The cGAS‐STING signaling pathway has garnered significant attention as a key innate immune pathway that plays a crucial role in tumor initiation and progression (Wei et al. 2024). In response to cellular perturbations, cyclic GMP‐AMP synthase (cGAS) recognizes cytoplasmic DNA from foreign microorganisms as well as mitochondrial DNA from damaged genomes (Du, Xu, and Cui 2021). The binding of cGAS to this double‐stranded DNA (dsDNA) induces the synthesis of the second messenger 2′3′‐cGAMP from ATP and GTP. This molecule binds to and activates STING, which is localized in the endoplasmic reticulum, thereby recruiting IRF3 and NF‐κB through TBK1 and IKK, respectively. The translocation of IRF3 and NF‐κB to the cell nucleus drives the expression of interferons (IFNs) and cytokines, ultimately inhibiting tumor initiation and development (Bose 2017; Gan et al. 2021). IFNs promote the activation and proliferation of immune cells, modulating both innate and adaptive immune responses to inhibit tumor progression (Zhou et al. 2020). Research has demonstrated that STING signaling is often suppressed in tumors (Ying‐Rui et al. 2023). In human gastric cancer (GC) tissues, STING expression significantly decreases in a TNM stage‐dependent manner and is inversely correlated with the prognosis of gastric cancer patients (Song et al. 2017). The anti‐tumor drug anlotinib can inhibit gastric cancer cell proliferation, migration, and immune escape by activating the cGAS‐STING signaling pathway (Yuan et al. 2022). Additionally, metformin activates the cGAS‐STING signaling pathway by inhibiting SOX2/AKT, offering potential for enhancing gastric cancer immunotherapy (Shen et al. 2023). Effective immune system activation relies on the use of STING agonists, and research on these agonists has gradually expanded with the discovery of numerous natural and synthetic compounds (Amouzegar et al. 2021). Previous studies have primarily focused on modifying endogenous cyclic dinucleotides (CDNs) such as cGAMP, c‐di‐AMP, and cyclic di‐GMP, all of which are well‐characterized STING agonists (Lu et al. 2023). A novel small molecule, M335, has recently been identified as a potent STING agonist (Zhao, Fan, et al. 2024). Research indicates that lipid nanoparticles loaded with STING agonists can mitigate anti‐PD‐1 resistance in melanoma lung metastasis through NK cell activation (Nakamura et al. 2021).

Activating the cGAS‐STING signaling pathway suppresses gastric cancer cell proliferation, migration, and immune evasion (Yuan et al. 2022). Moreover, STING agonists have exhibited potent anti‐tumor activity in various cancer types (Fang et al. 2023; Yin et al. 2022) and have been shown to overcome drug resistance (Nakamura et al. 2021; Huang et al. 2021). This prompts the question: can these agonists similarly mitigate cisplatin resistance in gastric cancer? Based on these premises and hypotheses, this study focused on the STING agonist c‐di‐AMP to explore its role in the progression of cisplatin‐resistant gastric cancer cells SGC‐7901/DDP.

### 1.3 Ginsenoside RG3 in Traditional Chinese Medicine

Ginseng has been utilized for health maintenance and the treatment of various ailments in Eastern countries for several millennia (Lu et al. 2021). Its primary active component is ginsenoside, with numerous ginsenosides having been studied and characterized (Choi 2008). Ginsenoside Rg3, a derivative of protopanaxadiol (PPD)‐type ginsenosides, undergoes partial removal of the sugar moiety, thereby enhancing its absorption in the human intestinal tract and augmenting its pharmacological activity (Shin and Oh 2016). Research has demonstrated that ginsenoside Rg3 exhibits a broad spectrum of medicinal properties, including antioxidant, anti‐aging, anti‐cancer, and immune‐enhancing effects. It is widely utilized in the treatment of cancer and the management of chemotherapy‐related side effects (De Costa et al. 2011; Guo, Kuruganti, and Gao 2019). Ginsenoside Rg3 promotes anti‐tumor immunity by modulating signal transduction pathways (Son et al. 2016). Studies indicate that Rg3 can activate various signaling pathways such as AMPK, JNK, NF‐κB, MAPKs, and the PI3K/AKT/mTOR axis, mediating pharmacological effects, including anti‐tumor activity, cardiovascular protection, immune regulation, neuroprotection, anti‐diabetic effects, anti‐fatigue, anti‐allergic responses, anti‐aging benefits, and antioxidant properties (Xu et al. 2023). Moreover, ginsenoside Rg3 can upregulate miR‐429 to inhibit SOX2 and the PI3K/Akt/mTOR signaling axis, thereby reducing cisplatin resistance in gastric cancer cells (Wang et al. 2022). Additionally, Rg3 enriches intestinal bacteria that produce acetate and propionate, inducing a host interferon (IFN‐I) response through the cGAS‐STING signaling axis, thereby providing both local and systemic protection against intestinal viral infections (Wang et al. 2023). Consequently, this study seeks to explore the effects of activating the cGAS‐STING signaling pathway in cisplatin‐resistant gastric cancer cells (SGC‐7901/DDP) using the STING agonist c‐di‐AMP in combination with the herbal medicine RG3. A series of in vitro and in vivo experiments have been developed to investigate the impact of this combined treatment on the progression of cisplatin‐resistant gastric cancer. The goal is to provide a theoretical foundation for the clinical management of cisplatin‐resistant gastric cancer and to identify new therapeutic targets.

### 1.4 Tumor Immunotherapy

In addition to traditional approaches such as surgery, radiation therapy, and chemotherapy, immunotherapy has emerged as a major cancer treatment modality and was named the top breakthrough of 2013 by *Science*. Tumor immunotherapy involves activating the body's own immune system to combat cancer and offers several advantages, such as treating various tumor types, targeting advanced‐stage tumors with metastasis, and preventing drug resistance, thus reducing recurrence rates (Rui, Zhou, and He 2023). As a highly researched field, numerous immunotherapy strategies are being investigated, with several showing substantial clinical efficacy. These strategies include immune checkpoint inhibitors (ICIs), adoptive cell therapy, and tumor vaccines. Under normal physiological conditions, immune cells can recognize and eliminate tumor cells. However, tumor cells can evade immune surveillance through various mechanisms, leading to immune escape, with immune checkpoints playing a crucial role in this process. Immunotherapy includes several strategies, such as adoptive cell therapy, tumor vaccines, oncolytic viruses, and ICIs. Currently, ICIs are a prominent focus of research. Prominent ICIs currently in clinical use are nivolumab, pembrolizumab, and atezolizumab, which have demonstrated effectiveness in treating various malignancies such as lung cancer, gastric cancer, and head and neck cancer (Naimi et al. 2022). The tumor microenvironment (TME) plays a critical role in the initiation and progression of tumors, primarily composed of blood vessels, immune cells, fibroblasts, and the extracellular matrix. These components interact to create an ecosystem conducive to tumor growth and progression (Arneth 2019). Tumor‐associated macrophages (TAMs) are a vital component of the TME and are closely linked to the occurrence, development, and metastasis of tumors (Pan et al. 2020).

This study seeks to explore the combined effects of the STING agonist c‐di‐AMP and the herbal compound ginsenoside RG3 in reversing cisplatin resistance in gastric cancer SGC‐7901/DDP cells. The goal is to identify new treatment targets for clinically drug‐resistant gastric cancer.

## 2 Materials and Methods

### 2.1 Cell Lines

The cisplatin‐resistant gastric cancer cell line SGC‐7901/DDP was obtained from the Tumor Research Center, along with human umbilical vein endothelial cells (HUVECs) and human lymphatic endothelial cells (HLECs). Cell experiments were organized into distinct groups: Control group (Control), STING agonist group (STING), Ginsenoside RG3 group (RG3), and the combined STING agonist and Ginsenoside RG3 group (STING agonist + RG3).

### 2.2 Western Blot Assay

Cell lysis was performed using RIPA buffer supplemented with protease and phosphatase inhibitors (CoWin Biosciences, Beijing, China). Subsequent protein quantification was performed using the BSA Protein Assay Kit (Roche, Basel, Switzerland). Polyacrylamide gel electrophoresis was used to separate proteins, followed by transfer to PVDF membranes (Millipore, Billerica, MA, USA). The PVDF membranes were incubated overnight at 4°C with the appropriate primary antibody, followed by a 1‐h incubation with the secondary antibody. Quantification and imaging procedures were performed using a gel imaging system (Bio‐RAD, Hercules, CA, USA).

### 2.3 MTT Assay

Cells were plated at a density of 3 × 10^3^ cells per well in 96‐well plates. Various treatments corresponding to experimental groups were administered at 0, 24, and 48 h. Following the treatments, cells were incubated with MTT (1 mg/mL) in 100 μL of medium per well for 4 h at 37°C. Subsequently, absorbance values were measured at 490 nm after the addition of 100 μL DMSO to each well.

### 2.4 Colony Formation Assay

Cells were plated at a density of 2 × 10^3^ cells per well in 6‐well plates and subjected to various treatments over a 14‐day period. Following incubation, cells were fixed with 4% paraformaldehyde for 30 min, washed with PBS, and stained with hematoxylin. The formed colonies were then analyzed.

### 2.5 Wound Healing Assay

Cells were seeded in six‐well plates and subjected to a scratch assay using 200 μL tips when the cells reached 80% confluence. Treatments were applied according to the experimental groups. Images were captured at 0, 24, and 48 h using an inverted microscope.

### 2.6 Transwell Assay

Cells were seeded at a density of 5 × 10^4^ cells per well in the upper chamber of a Transwell device (BD Biosciences, Piscataway, NJ, USA) and treated according to the respective experimental groups. The lower chamber was supplemented with DMEM containing 10% FBS. After a 48‐h incubation, cells were fixed with 4% paraformaldehyde, stained with hematoxylin, and observed under a microscope.

### 2.7 Immunofluorescence (IF) Assay

Cells were plated in six‐well plates and subjected to various treatments. Cells were fixed with 4% paraformaldehyde, permeabilized with 0.5% Triton‐X‐100, and blocked with 3% BSA (Solarbio, Beijing, China) for 2 h. Primary antibodies, including E‐cadherin (1:100), Vimentin (1:100), MDR1, MRP1 (1:200) (Santa Cruz Biotechnology, Dallas, TX, USA), and CD44 (1:200) (Cell Signaling Technology, USA), were incubated overnight at 4°C. The next day, secondary antibodies were applied for 2 h, and the cells were counterstained with DAPI (Solarbio, Beijing, China). Images were captured using a Leica SP5II laser confocal microscope.

### 2.8 Flow Cytometry Assay

SGC‐7901/DDP cells, at a concentration of 4 × 10^5^ cells per tube, were suspended in cell staining buffer. Cells were stained with propidium iodide (PI) (Biolegend, San Diego, USA) for 20 min at 4°C. After washing, cells were stained with fluorescein isothiocyanate (FITC) (Biolegend, San Diego, USA) for an additional 20 min at 4°C. The stained cells were analyzed using a BD Accuri flow cytometer (BD Biosciences, Piscataway, NJ, USA).

### 2.9 Endothelial Tube Formation Assay

A 96‐well plate was coated with 30 μL of Matrigel (BD Biosciences) and 30 μL of serum‐free RPMI‐1640 medium. Human umbilical vein endothelial cells (HUVECs) and human lymphatic endothelial cells (HLECs), each with a seeding density of 1 × 10^5^ cells per well, were plated onto the Matrigel‐coated plates. After a 6‐h incubation, images were captured using confocal laser microscopy.

### 2.10 In Vivo Tumorigenesis Assay

The animal study was approved by the Animal Ethics Committee of Yanbian University. Female Balb/c nude mice (aged 4–5 weeks) were obtained from Beijing Vital River Laboratory Animal Technology Co. Ltd. Mice were housed at 22°C ± 1°C with a 12‐h day/night cycle and had free access to food and water. Subcutaneous xenograft tumor models were established by injecting a mixture of 6 × 10^6^ SGC‐7901/DDP cells and Matrigel (BD Biosciences) into the fourth mammary fat pad of mice in each group. When the average tumor volume reached 150–200 mm^3^, mice were randomly divided into four groups: control, STING agonist, Rg3, and STING agonist + Rg3, with five mice per group. The control group received physiological saline, the STING agonist group received 25 μg STING agonist injected into the tumor mass, the Rg3 group was treated with 6 mg/kg/100 μL Rg3 via intraperitoneal injection, and the STING agonist + Rg3 group received both treatments. Treatments were administered every 3 days for 7 injections. After euthanasia, tumor, liver, kidney, and spleen tissues were collected and fixed in 10% formalin for subsequent hematoxylin and eosin (HE) staining or immunohistochemical analysis.

### 2.11 Ultrasound Imaging

The A Vevo2100 LAZR high‐frequency ultrasound (US) imaging system, manufactured by FUJIFILM VisualSonics Inc. in Toronto, Ontario, Canada, served as the platform for all US image acquisitions. This system utilized a linear array transducer (LZ‐550) with a center frequency ranging from 32 to 55 MHz. Employing fiber‐optic transducers, the system delivered nanosecond laser pulses into deep anatomical targets. The photoacoustic and spatial dimensions of the US images were confined within 14 mm in width and under 15 mm in depth. Image capture occurred at three‐day intervals, totaling seven times in all, once the average tumor volume reached 150–200 mm^3^.

### 2.12 Immunohistochemistry Staining

Tumor tissue was immersed in 4% paraformaldehyde for 24 h for proper fixation. After fixation, adequately sized tissue samples were transferred into embedding boxes for dehydration and subsequent embedding. The microtome was activated, securing the pre‐cooled wax block in a 4°C refrigerator. The blade's distance from the wax block was adjusted and the slice thickness was set to 4 μm before initiating the slicing process. Then we proceeded to de‐paraffinize and rehydrate tissue sections for antigenic thermal repair, allowing natural cooling at room temperature. Endogenous peroxidase activity was counteracted by incubating sections in 3% hydrogen peroxide (H_2_O_2_) (ZSGB‐BIO) for 30 min. The tissue sections were incubated overnight at 4°C with the respective primary antibodies (MDR1, MRP1, E‐Cadherin, Vimentin, Ki67, CD44). On the following day, wet cassettes were prewarmed at 37°C for 1 h, and then the sections were immersed in a PBS rinse within a small dye vat. The sections were incubated with secondary antibodies, diluted in PBS (protected from light), in a molecular hybridization chamber at 37°C for 1 h. Subsequently, wash sections with PBS three times for 5 min each. Conclude the staining process by applying 3,3′‐diaminobenzidine (DAB) (ZSGB‐BIO) and hematoxylin solution to tissue sections in succession.

### 2.13 HE Staining

In the preparation of tissue sections, deparaffinization was carried out using xylene I and II for 10 min each, with glass slides prepared in advance. Subsequently, the sections were immersed in 100% (I and II), 90%, 80%, and 70% ethanol for 5 min each, followed by a thorough rinse with tap water for 5 min (repeated three times). Staining was performed using hematoxylin for 5 min, and staining time was adjusted based on the observed intensity. The sections were rinsed under running water. Differentiation was achieved with 5% acetic acid for 1 min, followed by water rinsing. Acetic acid was added dropwise using a pipette until tissue coverage was complete, resulting in a lighter, bluish coloration. Counterstaining was done with eosin for 1 min, adjusting the staining time as needed and then rinsed under running water. Dehydration was carried out in 70%, 80%, 90%, and 100% ethanol for 10 s each, followed by xylene for 1 min. Finally, the sections were sealed in a fume hood.

### 2.14 Statistics

Statistical analysis was performed using GraphPad Prism 8.0 software (GraphPad, La Jolla, CA, USA). Two‐group comparisons were conducted using Student's t‐test, and multiple group comparisons were assessed through One‐way ANOVA. A *p* value less than 0.05 was considered statistically significant (*p < 0.05, **p < 0.01, ***p < 0.001, ****p < 0.0001), while a *p* value greater than 0.05 indicated no statistically significant difference (ns). To ensure consistency and reliability, experiments were replicated three times.

## 3 Results

### 3.1 Synergistic Inhibition of SGC‐7901/DDP Cell Proliferation, Migration, and Angiogenesis by STING Agonist and Ginsenoside RG3

#### 3.1.1 c‐di‐AMP Effectively Activates the cGAS‐STING Signaling Pathway in SGC‐7901/DDP Cells

**FIGURE 1:**
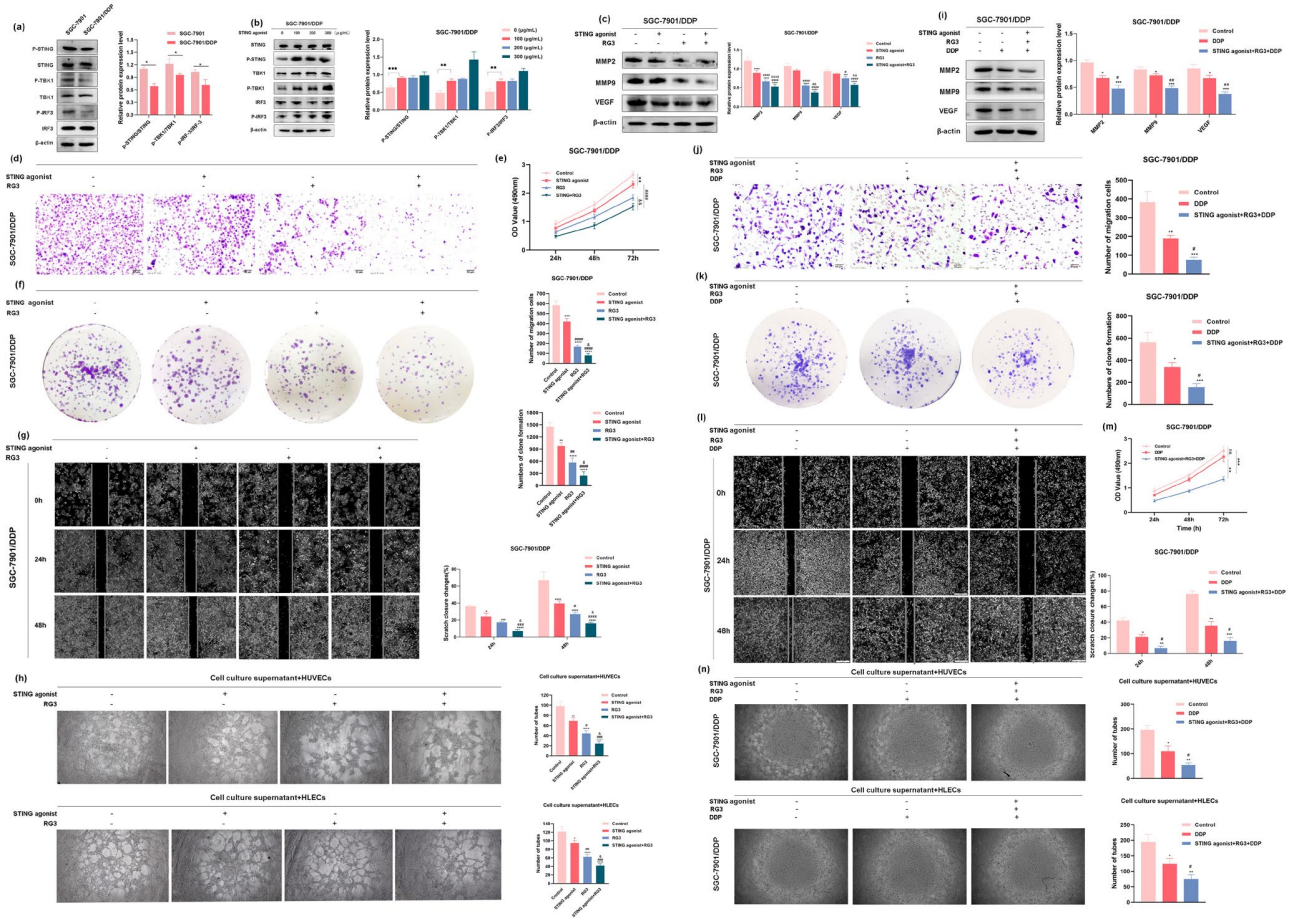
STING agonist and RG3 inhibit proliferation, migration, invasion, and tube formation in SGC‐7901/DDP cells and enhance sensitivity to Cisplatin. (a) Differential expression of cGAS‐STING signaling pathway‐related proteins in SGC‐7901 and SGC‐7901/DDP cells. (b) Gastric cancer cells SGC‐7901/DDP were treated with different concentrations (0, 100, 200, and 300 μg/mL) of c‐di‐AMP for 48 h, followed by Western blot analysis to assess the expression of STING, p‐STING, TBK1, P‐TBK1, IRF3, and P‐IRF3. (c) Western blot analysis evaluated the impact of STING agonist and RG3 treatment on the expression of VEGF, MMP2, and MMP9 proteins in SGC‐7901/DDP cells. (d) Transwell assays were conducted to observe the effect of STING agonist and RG3 on the vertical migration of SGC‐7901/DDP cells. (e) MTT assay was performed to assess the influence of STING agonist and RG3 treatment on the proliferation capacity of SGC‐7901/DDP cells. (f) Impact of STING agonist and RG3 on the colony‐forming ability of SGC‐7901/DDP cells, evaluated through colony formation assays. (g) Scratch assays were employed to assess the effect of STING agonist and RG3 treatment on the wound healing ability of SGC‐7901/DDP cells. (h) Evaluation of the impact of STING agonist and RG3 on the tubulogenesis ability of gastric cancer cells. Endothelial cell tube formation assays detected microvessel formation in HUVECs (human umbilical vein endothelial cells) and HLECs (human lymphatic endothelial cells) treated with conditioned medium. (i) Western blot analysis evaluated the impact of DDP and STING agonist + RG3 + DDP treatment on the expression of VEGF, MMP2, and MMP9 proteins in SGC‐7901/DDP cells. (j) Transwell assays were conducted to observe the effect of DDP and STING agonist + RG3 + DDP on the vertical migration of SGC‐7901/DDP cells. (k) Impact of DDP and STING agonist + RG3 + DDP on the colony‐forming ability of SGC‐7901/DDP cells, evaluated through colony formation assays. (l) Scratch assays were employed to assess the effect of DDP and STING agonist + RG3 + DDP treatment on the wound healing ability of SGC‐7901/DDP cells. (m) MTT assay was performed to assess the influence of DDP and STING agonist+RG3 + DDP treatment on the proliferation capacity of SGC‐7901/DDP cells. (n) Evaluation of the impact of DDP and STING agonist + RG3 + DDP on the tubulogenesis ability of gastric cancer cells. Endothelial cell tube formation assays detected microvessel formation in HUVECs and HLECs treated with conditioned medium. (* denotes comparison with the control group. # represents comparison with the STING agonist group or DDP group. & denotes comparison with the RG3 group. *, #, & indicate statistical significance (*p <* 0.05). **, ##, && indicate statistical significance at *p <* 0.01. ***, ###, &&& indicate statistical significance at *p <* 0.001. ****, #### indicate statistical significance at *p <* 0.0001).

We performed western blot experiments to evaluate the expression of proteins associated with the STING signaling pathway, including STING, phosphorylated STING (pSTING), TBK1, phosphorylated TBK1 (pTBK1), IRF3, and phosphorylated IRF3 (pIRF3), in both the parental gastric cancer cell line SGC‐7901 and its cisplatin‐resistant counterpart SGC‐7901/DDP. The results indicated that STING expression was marginally higher in SGC‐7901 compared to SGC‐7901/DDP, with no significant differences observed in the expression of TBK1 and IRF3 between the two cell lines. Notably, the levels of pSTING, pTBK1, and pIRF3 were reduced in the cisplatin‐resistant SGC‐7901/DDP cells relative to the parental SGC‐7901 cells (Figure 1a). This suggests that the development of cisplatin resistance in gastric cancer cells may be linked to the inactivation of the STING signaling pathway. Therefore, further investigation into the mechanisms underlying STING pathway inactivation and strategies to activate this pathway is crucial for enhancing the sensitivity of gastric cancer cells to cisplatin, providing new therapeutic strategies for overcoming chemotherapy resistance in gastric cancer. Subsequently, SGC‐7901/DDP cells were treated with c‐di‐AMP at concentrations of 0, 50, 100, and 200 μg/mL for 48 h. Protein extraction and Western blot analyses were subsequently performed to evaluate the expression of STING, p‐STING, TBK1, and p‐TBK1. The results indicated that c‐di‐AMP at a concentration of 100 μg/mL effectively upregulated the expression of p‐STING and p‐TBK1, thereby activating the cGAS‐STING signaling pathway in SGC‐7901/DDP cells (Figure 1b). Consequently, this concentration of c‐di‐AMP was chosen for subsequent treatments.

In our previous investigation (Ziqi et al. 2022), the IC50 of cisplatin (DDP) for SGC‐7901 cells was determined to be 0.65 μg/mL, whereas for SGC‐7901/DDP cells, it was 1.40 μg/mL. This indicated an enhanced resistance of SGC‐7901/DDP cells to cisplatin. MTT assay results further indicated that the viability of SGC‐7901/DDP cells in the co‐treatment group, which included 40 μg/mL of ginsenoside Rg3 and varying concentrations of DDP (0, 0.2, 0.4, 0.8, 1.6, and 3.2 μg/mL), was significantly lower than that of the control group. Therefore, this dosage was chosen for subsequent experiments involving ginsenoside Rg3.

#### 3.1.2 STING Agonist Cooperates With Ginsenoside RG3 to Inhibit SGC‐7901/DDP Cell Proliferation

To evaluate the inhibitory effects of the combined treatment with a STING agonist and RG3 on SGC‐7901/DDP cells, both MTT assays and colony formation assays were performed. The experimental groups comprised control, STING agonist, RG3, and STING agonist + RG3. MTT assay results (Figure 1e) revealed a reduced proliferation capacity of cells in both the STING agonist and RG3 groups relative to the control group. Notably, the combined treatment with the STING agonist and RG3 exhibited a more pronounced inhibitory effect on the proliferation of SGC‐7901/DDP cells. Consistent results were observed in the colony formation assay, where the combination treatment significantly reduced the colony‐forming ability of SGC‐7901/DDP cells relative to the control group (Figure 1f). These collective findings strongly indicate that the synergistic action of the STING agonist and RG3 effectively inhibits the proliferation of SGC‐7901/DDP cells.

#### 3.1.3 Combined Inhibition of SGC‐7901/DDP Cell Migration by STING Agonist and Ginsenoside RG3

We conducted cell scratch assays, transwell assays, and western blot experiments to evaluate the impact of the STING agonist and RG3 on the migration capacity of SGC‐7901/DDP cells. The results of the scratch assay (Figure 1g) revealed that, compared to the control group, the lateral migration capacity of SGC‐7901/DDP cells was significantly inhibited in all three treatment groups. Notably, the group treated with the combination of the STING agonist and RG3 exhibited a more pronounced inhibitory effect on lateral migration of SGC‐7901/DDP cells. Transwell assay results (Figure 1d) further revealed that, relative to the control group, the STING agonist, RG3, and combination treatment groups all impeded the vertical migration capacity of SGC‐7901/DDP cells. Among these, the combination treatment with the STING agonist and RG3 significantly suppressed the vertical migration of SGC‐7901/DDP cells.

Matrix metalloproteinases (MMPs) constitute a subclass of endopeptidases within the extracellular matrix, playing a crucial role in physiological processes such as organ development, angiogenesis, apoptosis, and cellular motility (Xie et al. 2017). Conversely, they play a pathological role in the initiation and progression of cancer, contributing to tumor degradation, neovascularization, and subsequent metastasis (Quintero‐Fabián et al. 2019). Within the MMP family, MMP‐2 and MMP‐9 are particularly significant, as their release by cancer cells facilitates matrix degradation, thereby promoting infiltration, invasion, and distant metastasis.

In this study, western blot experiments were performed to assess the expression of MMP2 and MMP9 proteins in SGC‐7901/DDP cells following various treatments (Figure 1c). The findings revealed that, compared to the control group, the expression of migration‐related proteins in the STING agonist group decreased, with statistical significance observed only for MMP2. Both the RG3 group and the combination treatment group exhibited significantly reduced expression of MMP2 and MMP9, with the combination treatment showing a more pronounced inhibitory effect.

#### 3.1.4 Synergistic Inhibition of SGC‐7901/DDP Cell Angiogenesis by STING Agonist and Ginsenoside RG3

Angiogenesis plays a pivotal role in tumor initiation and progression by supplying growing neoplasms with essential oxygen and nutrients (Cheng et al. 2023). In oncology, inhibiting pathological angiogenesis has been widely recognized as an effective therapeutic strategy for cancer treatment (Griffioen and Dudley 2022). To further investigate whether the combination of the STING agonist and RG3 can inhibit angiogenesis in SGC‐7901/DDP cells, we performed a tube formation assay. The results (Figure 1h) demonstrated that, compared to the control group, all three treatment groups significantly inhibited the tube formation ability of human umbilical vein endothelial cells (HUVECs) and human lymphatic endothelial cells (HLECs) after 6 h of exposure to the supernatant from SGC‐7901/DDP cells, with statistical significance observed. Notably, the combination treatment group with the STING agonist and RG3 exhibited a more pronounced inhibitory effect.

Vascular endothelial growth factor (VEGF) is a key regulatory factor in both normal and tumor angiogenesis (Bokhari and Hamar 2023). We assessed VEGF expression in SGC‐7901/DDP cells following different treatments using western blot analysis (Figure 1c). The results indicated that, compared to the control group, the change in VEGF expression in the STING agonist group was not statistically significant. However, both the RG3 group and the combination treatment group with the STING agonist and RG3 showed significant downregulation of VEGF expression, with a more pronounced effect observed in the combination treatment group.

These findings collectively suggest that the synergistic effect of the STING agonist and RG3 significantly inhibits both lateral and vertical migration abilities, tube formation capacity, and downregulates the expression of metastasis‐related proteins MMP‐2 and MMP‐9, as well as the angiogenesis‐related protein VEGF. Consequently, this combined treatment effectively suppresses the malignant progression of SGC‐7901/DDP cells.

#### 3.1.5 The Synergistic Effect of Ginsenoside RG3 and the STING Agonist Enhances Cisplatin Sensitivity in SGC‐7901/DDP Cells

To investigate the effect of ginsenoside RG3 combined with a STING agonist on cisplatin sensitivity in SGC‐7901/DDP cells, we categorized the SGC‐7901/DDP cells into the following groups: a control group, a cisplatin‐treated group (10 μM DDP) (Cao et al. 2021; Zhao et al. 2023), and a STING agonist + RG3 + DDP group. We comprehensively evaluated the capacity of the STING agonist combined with RG3 to overcome cisplatin resistance in SGC‐7901/DDP cells through Western blot, MTT, colony formation, wound healing, transwell, and tube formation assays.

Matrix metalloproteinases (MMPs) are extracellular matrix endopeptidases essential for tissue formation, apoptosis, and angiogenesis. Western blot results revealed that DDP downregulated MMP2 and MMP9 expression compared to the control group, with a more pronounced effect observed in the STING agonist + RG3 + DDP group (Figure 1i). MTT and colony formation assays showed that DDP inhibited SGC‐7901/DDP cell proliferation, with the inhibitory effect significantly enhanced in the STING agonist + RG3 + DDP group (Figure 1k,m). Wound healing and transwell assays revealed significant suppression of SGC‐7901/DDP cell horizontal and vertical migration in the STING agonist + RG3 + DDP group, with a more pronounced effect than DDP alone (Figure 1j,l). Vascular endothelial growth factor (VEGF), a key regulator of angiogenesis, was assessed using western blot in treated SGC‐7901/DDP cells. The results demonstrated significant VEGF downregulation in the STING agonist + RG3 + DDP group compared to the control group (Figure 1i). To confirm the anti‐angiogenic effect of the STING agonist combined with RG3, we assessed tube formation ability. The results showed that tube formation was inhibited in the combination treatment group compared to the control group, with a more pronounced effect than DDP treatment alone (Figure 1n).

In conclusion, the STING agonist combined with RG3 enhances cisplatin sensitivity in SGC‐7901/DDP cells by inhibiting their proliferation, migration, and tube formation, effectively reversing cisplatin resistance in gastric cancer cells.

### 3.2 Induction of Apoptosis in SGC‐7901/DDP Cells by Synergistic Action of STING Agonist and Ginsenoside RG3

#### 3.2.1 Formation of Apoptotic Bodies Induced by the Synergistic Action of STING Agonist and Ginsenoside RG3 in SGC‐7901/DDP Cells

**FIGURE 2:**
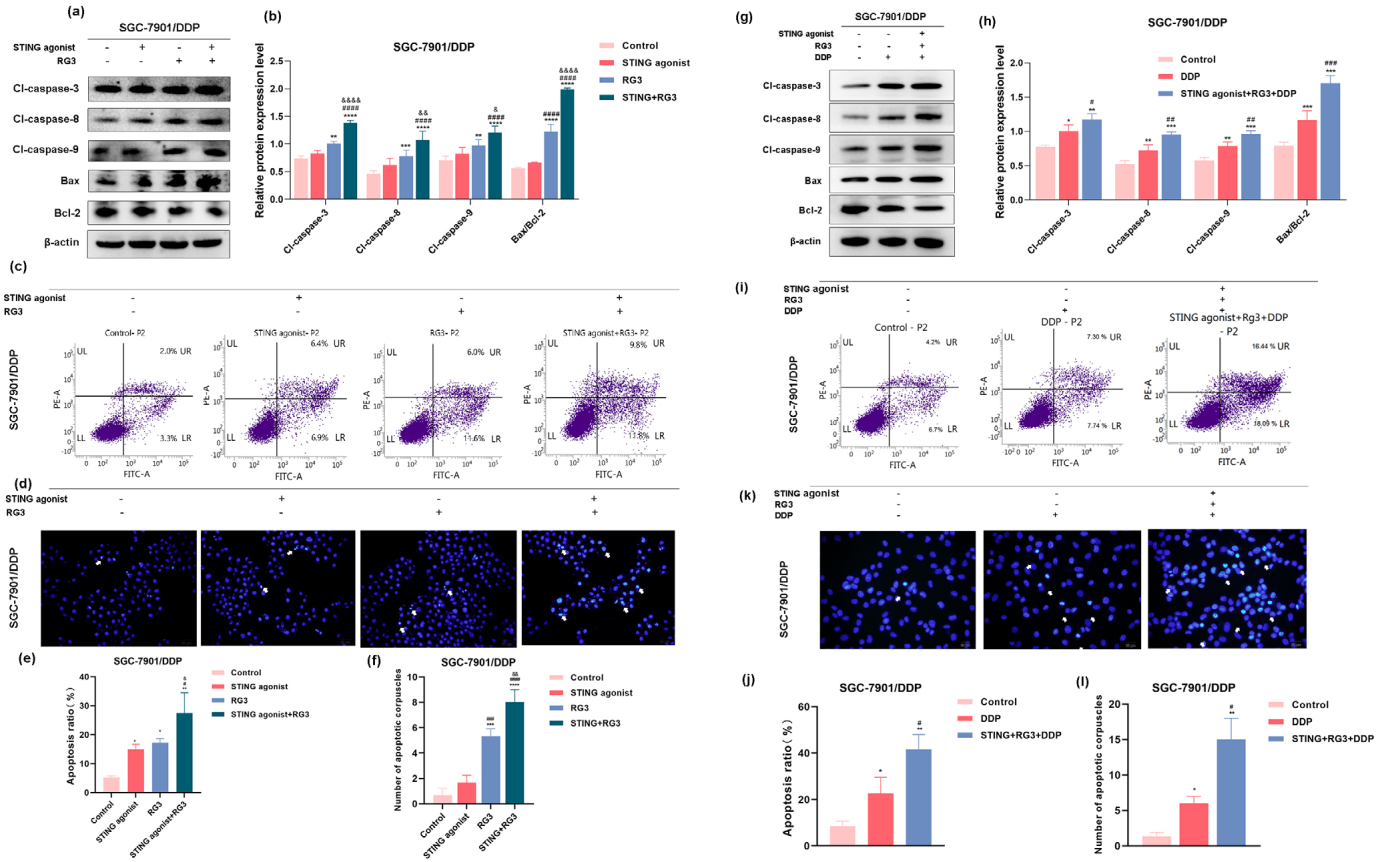
Impact of STING agonist and RG3 on the apoptotic function of gastric cancer cells. (a and b) Western blot analysis assessing the influence of STING agonist and RG3 on the expression of apoptotic‐related proteins in gastric cancer cells. (c, e) Flow cytometry used to evaluate the induction of apoptosis in SGC‐7901/DDP cells following treatment with STING agonist and RG3. (d, f) Hoechst 33342 staining depicting the morphological changes induced by STING agonist and RG3 treatment in apoptotic SGC‐7901/DDP cells. (g, h) Western blot analysis assessing the influence of DDP and STING agonist+RG3 + DDP on the expression of apoptotic‐related proteins in gastric cancer cells. (i, j) Flow cytometry used to evaluate the induction of apoptosis in SGC‐7901/DDP cells following treatment with DDP and STING agonist+RG3 + DDP. (k, l) Hoechst 33342 staining depicting the morphological changes induced by DDP and STING agonist + RG3 + DDP treatment in apoptotic SGC‐7901/DDP cells. (* denotes comparison with the control group. # represents comparison with the STING agonist group or DDP group. & denotes comparison with the RG3 group. *, #, & indicate statistical significance at *p <* 0.05. **, ##, && indicate statistical significance at *p <* 0.01. ***, ###, &&& indicate statistical significance at *p <* 0.001. ****, #### indicate statistical significance at *p <* 0.0001).

Hoechst 33342 is widely recognized for its ability to penetrate intact cell membranes and intercalate into nuclear DNA, emitting characteristic blue fluorescence. This post‐staining method highlights nuclear chromatin in apoptotic cells, yielding a more intense, circular, or condensed clustered structure. It is frequently used to observe cellular morphology and identify apoptotic cells (Yang, Li, et al. 2023). To evaluate the effect of the STING agonist and RG3 on apoptosis in SGC‐7901/DDP cells, we employed Hoechst 33342 staining to observe treatment‐induced changes in cellular morphology. The results (Figure 2d,f) showed that in the Control group, SGC‐7901/DDP cells displayed intact morphology and relatively uniform blue fluorescence. Compared to the Control group, the STING agonist group showed a slight increase in apoptotic bodies, which was not statistically significant. In contrast, the RG3 group and the STING agonist + RG3 combination treatment group exhibited a significant increase in bright blue apoptotic bodies, characterized by distinct apoptotic features, including nuclear condensation and fragmentation. These changes were statistically significant, with the most pronounced increase observed in the STING agonist + RG3 combination treatment group.

#### 3.2.2 STING Agonist and Ginsenoside RG3 Synergistically Induce Apoptosis in SGC‐7901/DDP Cells

To investigate the effects of STING agonist and RG3 treatment on apoptosis in SGC‐7901/DDP cells, we used Annexin V‐FITC/PI double staining to evaluate apoptotic responses under various treatment conditions (Figure 2c,e). The results indicated that apoptotic rates in SGC‐7901/DDP cells significantly increased in all three treatment groups compared to the control group, with statistical significance. Notably, the combination treatment group of the STING agonist and RG3 showed a more substantial increase in apoptotic rates, suggesting a synergistic effect in apoptosis induction.

Caspases are proteases that cleave substrates and are categorized into two groups: inflammatory caspases, which regulate inflammation, and apoptotic caspases, which mediate apoptosis. Apoptotic caspases are crucial in programmed cell death and are divided into initiator caspases (Caspase‐8, 9, and 10) and executioner caspases (Caspase‐3, 6, and 7) (Green 2022). Western blot analysis was subsequently conducted to measure the expression levels of apoptosis‐related proteins, including Caspase‐3, Caspase‐8, and Caspase‐9 (Kariya, Gu, and Kariya 2023) (Figure 2a,b), as well as the pro‐apoptotic protein Bax and the anti‐apoptotic protein Bcl‐2. An elevated Bax/Bcl‐2 ratio is indicative of apoptosis promotion. The results showed that apoptotic protein expression increased in the STING agonist group compared to the control group, though this change was not statistically significant. However, both the RG3 group and the combination treatment group of the STING agonist and RG3 showed elevated apoptotic protein expression and Bax/Bcl‐2 ratios, with the combination group exhibiting the greatest increase.

In summary, the combined action of the STING agonist and RG3 significantly enhances apoptotic body formation and increases the apoptotic rate in SGC‐7901/DDP cells. This effect is mediated through the induction of apoptosis via both the death receptor and mitochondrial pathways.

#### 3.2.3 The Combination of the STING Agonist and RG3 Enhances Cisplatin Sensitivity in SGC‐7901/DDP Cells, Leading to Increased Apoptosis

We performed additional experiments to investigate how ginsenoside RG3 combined with a STING agonist influences cisplatin sensitivity in SGC‐7901/DDP cells, focusing on apoptotic function. SGC‐7901/DDP cells were divided into three groups: control, cisplatin (DDP), and STING agonist + RG3 + DDP (cisplatin concentration of 10 μM). The effects of STING agonist, RG3, and DDP treatments on apoptosis in SGC‐7901/DDP cells were evaluated using Western blot, flow cytometry, and Hoechst 33342 staining. Western blot analysis showed that, compared to the control group, the expression of apoptosis‐related proteins Caspase‐3, Caspase‐8, Caspase‐9, and the pro‐apoptotic protein Bax significantly increased in both the DDP and STING agonist + RG3 + DDP groups. The upregulation of these proteins was most pronounced in the combination treatment group (Figure 2g,h). Flow cytometry was then performed to evaluate the apoptosis ratio in SGC‐7901/DDP cells following treatment. The results indicated that the apoptosis ratio increased in the DDP group compared to the control group, with a more significant increase observed in the STING agonist + RG3 + DDP group (Figure 2i,j). Hoechst 33342 staining was performed to detect apoptotic body formation in the nuclei of SGC‐7901/DDP cells. The results showed that, compared to the control group, distinct bright blue apoptotic bodies were observed in the nuclei of the DDP group, with the greatest number observed in the combination treatment group (Figure 2k,l; arrows indicate apoptotic bodies). In conclusion, combining the STING agonist with RG3 enhances the sensitivity of SGC‐7901/DDP cells to cisplatin, resulting in increased apoptosis.

### 3.3 STING Agonist and Ginsenoside RG3 Synergistically Inhibit the EMT Process in SGC‐7901/DDP Cells

Epithelial‐mesenchymal transition (EMT) plays a pivotal role in tumor progression, regulated by gene expression, and contributes to cancer invasion and metastasis while generating cells with stem cell‐like characteristics (Neumann et al. 2024; Lüönd et al. 2021). Studies have indicated a correlation between cell stemness, drug resistance, and EMT (Pan et al. 2021). To investigate the impact of the STING agonist and RG3 on the EMT process in SGC‐7901/DDP cells, we conducted validation through Western blot experiments and immunofluorescence assays.

**FIGURE 3:**
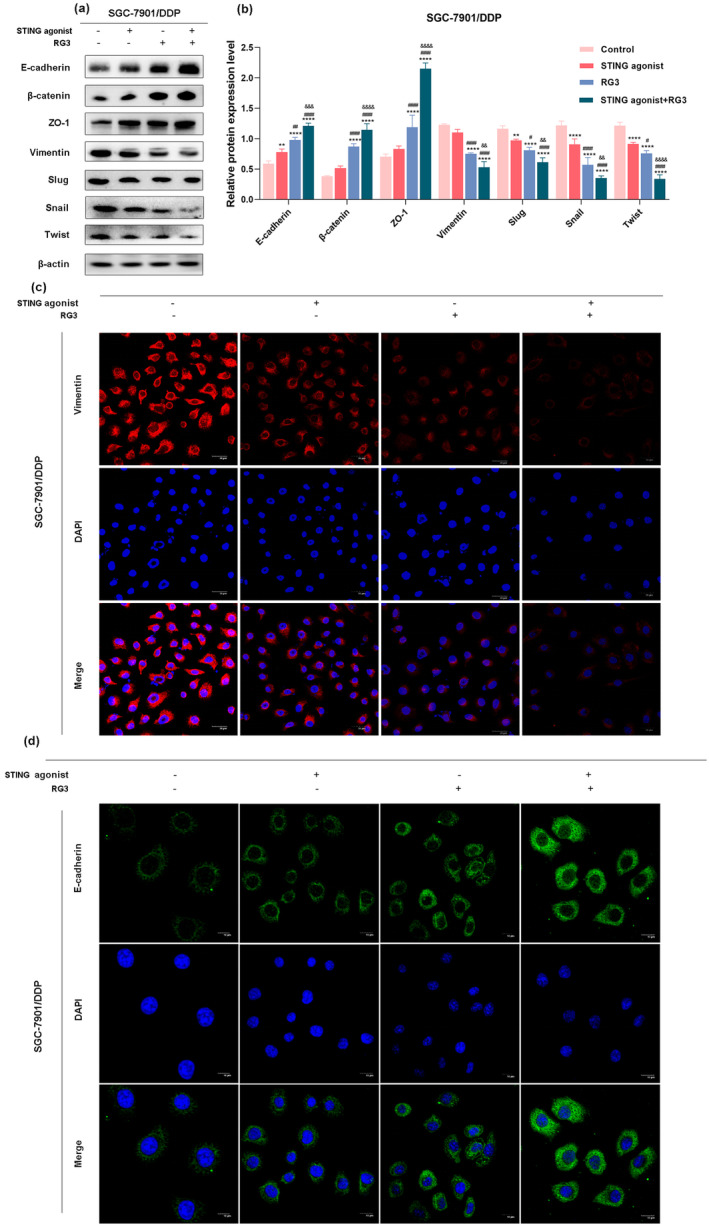
Impact of STING agonist and RG3 on the Epithelial‐Mesenchymal Transition (EMT) process in gastric cancer. (a and b) Western blot analysis assessing the expression of EMT‐related proteins in SGC‐7901/DDP cells following treatment with STING agonist and RG3. (c and d) Immunofluorescence evaluation of the fluorescence signal intensity of E‐cadherin and Vimentin in SGC‐7901/DDP cells after treatment with STING agonist and RG3. (* denotes comparison with the control group. # represents comparison with the STING agonist group. & denotes comparison with the RG3 group. *, #, & indicate statistical significance at *p <* 0.05. **, ##, && indicate statistical significance at *p <* 0.01. ***, ###, &&& indicate statistical significance at *p <* 0.001. ****, #### indicate statistical significance at *p <* 0.0001).

The western blot results (Figure 3a,b) demonstrated that, compared to the control group, the expression levels of epithelial markers (E‐cadherin, β‐catenin, ZO‐1) increased, while mesenchymal markers (Vimentin, Slug, Snail, Twist) were suppressed in the other three treatment groups. However, in the STING agonist group, these expression changes were only partially statistically significant. In both the RG3 group and the combination treatment group with the STING agonist and RG3, the changes in marker protein expression were statistically significant, with a more pronounced effect observed in the combination treatment group.

Immunofluorescence assay results (Figure 3c,d) showed that, compared to the control and STING agonist groups, the fluorescence signal of E‐Cadherin significantly increased, while the signal for Vimentin decreased in the RG3 group and the combination treatment group. In the combination treatment group, the fluorescence signal changes for both markers were more pronounced, indicating a more significant inhibitory effect on EMT when the STING agonist and RG3 were used together.

Collectively, these data suggest that the combined use of the STING agonist and RG3 inhibits the EMT process in SGC‐7901/DDP cells, thereby suppressing their malignant progression.

### 3.4 STING Agonist and Ginsenoside RG3 Synergistically Suppress Stemness in SGC‐7901/DDP Cells

**FIGURE 4:**
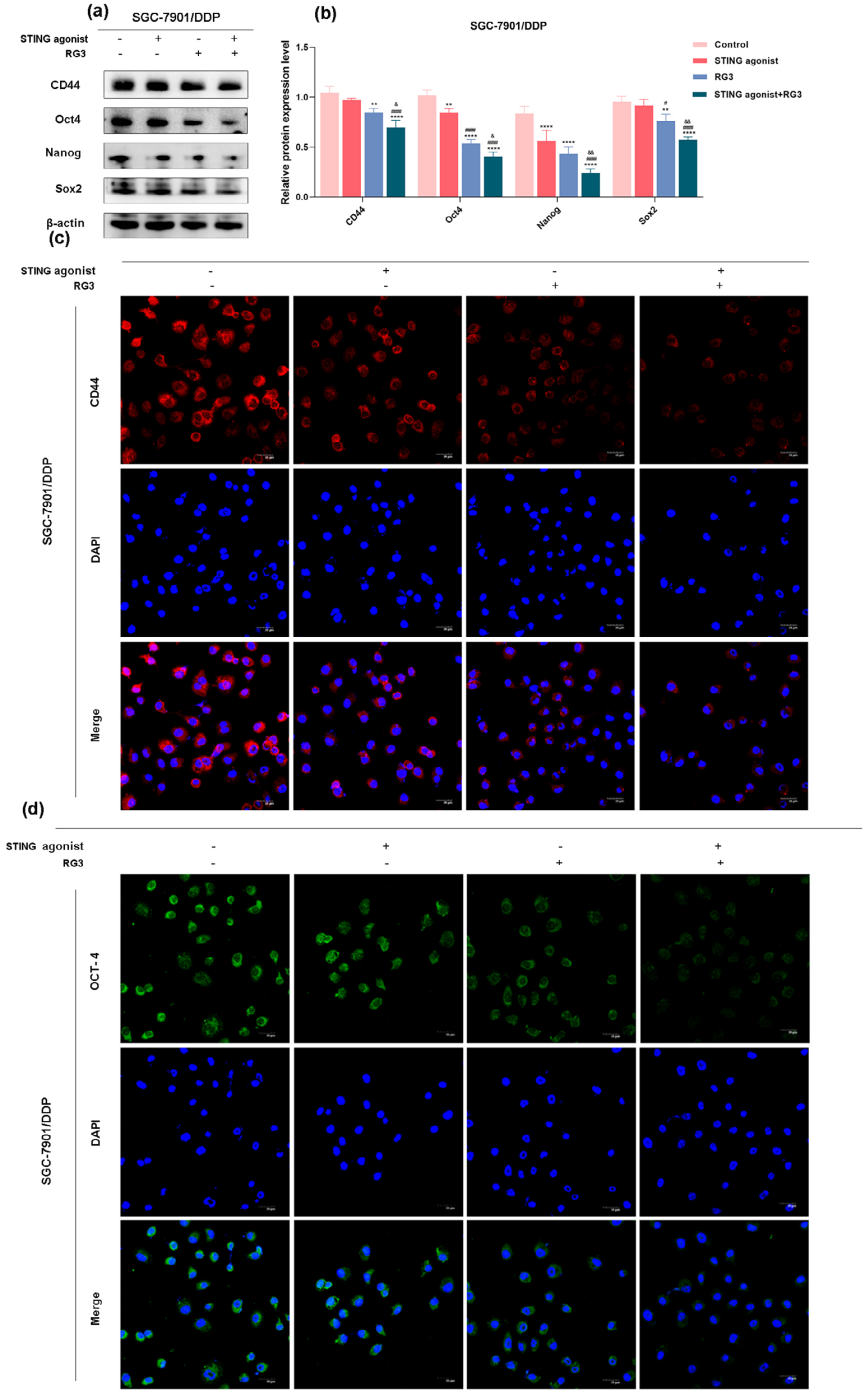
Inhibitory effect of the combined use of STING agonist and RG3 on the stemness of gastric cancer cells. (a and b) Western blot analysis of the expression of stemness‐related proteins in SGC‐7901/DDP cells after treatment with STING agonist and RG3. (c and d) Immunofluorescence examination of the fluorescence intensity changes of the stemness marker CD44 in SGC‐7901/DDP cells following treatment with STING agonist and RG3. (* denotes comparison with the control group. # represents comparison with the STING agonist group. & denotes comparison with the RG3 group. *, #, & indicate statistical significance at *p <* 0.05. **, ##, && indicate statistical significance at *p <* 0.01. ***, ###, &&& indicate statistical significance at *p <* 0.001. ****, #### indicate statistical significance at *p <* 0.0001).

Stemness, defined as the ability of cancer cells to sustain differentiation (Malta et al. 2018), was assessed using western blot and immunofluorescence assays. Western blot analysis revealed that, compared to the control group, the expression levels of stemness markers (CD44, Oct4, Nanog, and Sox2) were reduced in the other three treatment groups. Notably, in the STING agonist group, these reductions were only partially statistically significant. In contrast, both the RG3 group and the STING agonist + RG3 combination treatment group exhibited statistically significant reductions in the expression of these marker proteins, with the most pronounced effect observed in the combination treatment group (Figure 4a,b). Immunofluorescence assay results indicated that, compared to the control and STING agonist groups, the fluorescence signals of CD44 and Oct4 significantly decreased in the RG3 group and the STING agonist + RG3 combination treatment group, with the most substantial reduction observed in the combination treatment group (Figure 4c,d). These findings suggest that the STING agonist, in combination with ginsenoside RG3, effectively suppresses the stemness of SGC‐7901/DDP cells.

### 3.5 STING Agonist and Ginsenoside RG3 Synergistically Reverse Cisplatin Resistance in SGC‐7901/DDP Cells

**FIGURE 5:**
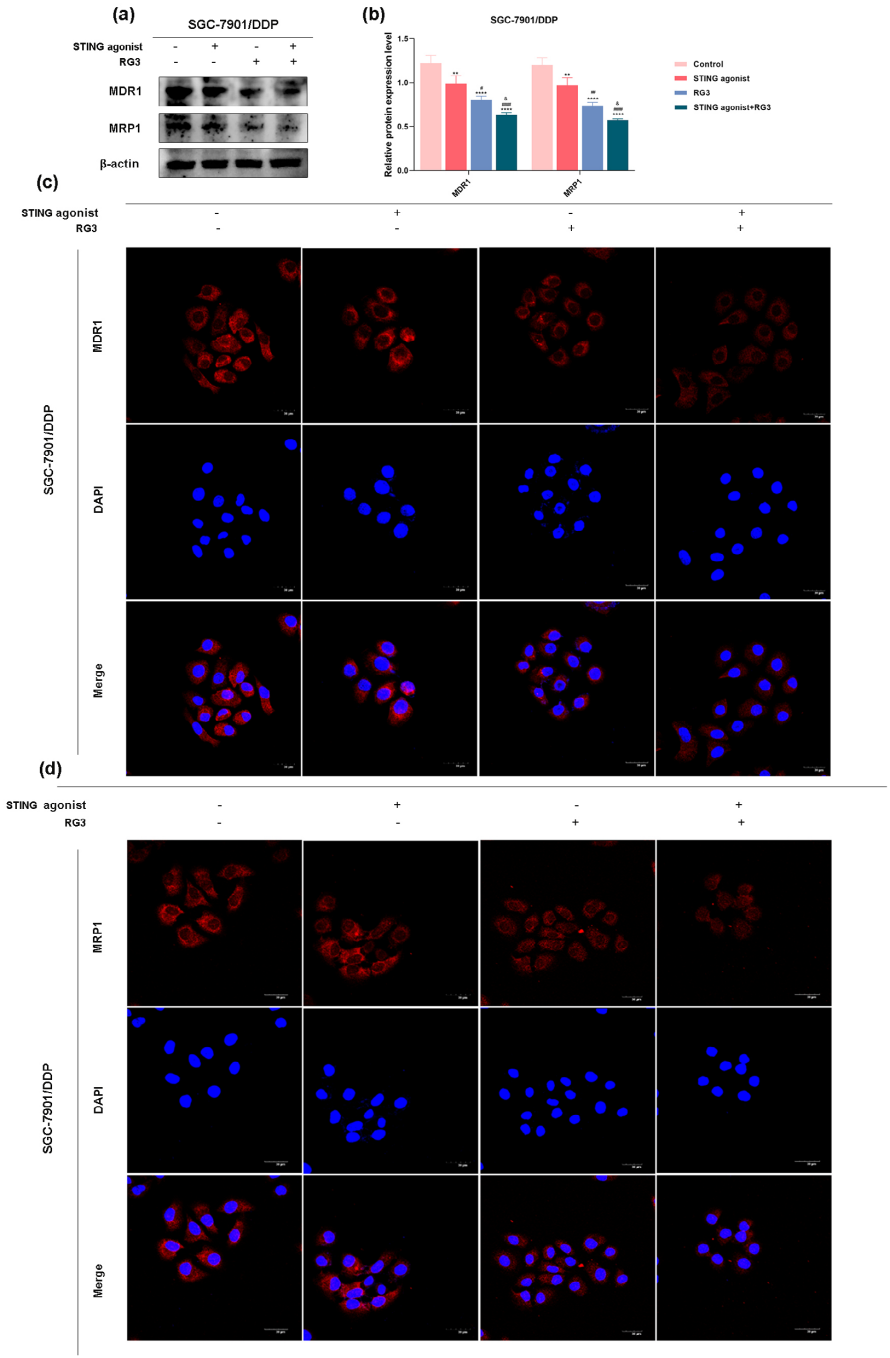
Reversal of cisplatin resistance in gastric cancer cells by the combined use of STING agonist and RG3. (a and b) Western blot analysis showing the expression levels of resistance proteins MDR1 and MRP1. (c and d) Immunofluorescence detection of the fluorescence intensity of resistance proteins MDR1 and MRP1. (* denotes comparison with the control group. # represents comparison with the STING agonist group. & denotes comparison with the RG3 group. *, #, & indicate statistical significance at *p <* 0.05. **, ##, && indicate statistical significance at *p <* 0.01. ***, ###, &&& indicate statistical significance at *p <* 0.001. ****, #### indicate statistical significance at *p <* 0.0001).

Cisplatin resistance in gastric cancer is characterized by the progressive loss of sensitivity to cisplatin, a chemotherapy agent, during treatment, ultimately reducing the effectiveness of the therapy. To explore whether the combined use of the STING agonist and RG3 can synergistically overcome cisplatin resistance in SGC‐7901/DDP cells, Western blot experiments were performed. The results indicated that, compared to the control group, the expression levels of resistance markers MDR1 and MRP1 were significantly reduced in the other three treatment groups, with the most pronounced effect observed in the combination treatment group (Figure 5a,b). Immunofluorescence assay results corroborated the western blot findings. Relative to the control group and the groups treated with the STING agonist or RG3 alone, the fluorescence signals of MDR1 and MRP1 were significantly diminished in the combination treatment group (Figure 5c,d). These findings suggest that the STING agonist, in combination with RG3, effectively reverses cisplatin resistance in SGC‐7901/DDP cells.

### 3.6 Synergistic Inhibition of Gastric Cancer Progression by STING Agonists and Ginsenoside Rg3 In Vivo

**FIGURE 6:**
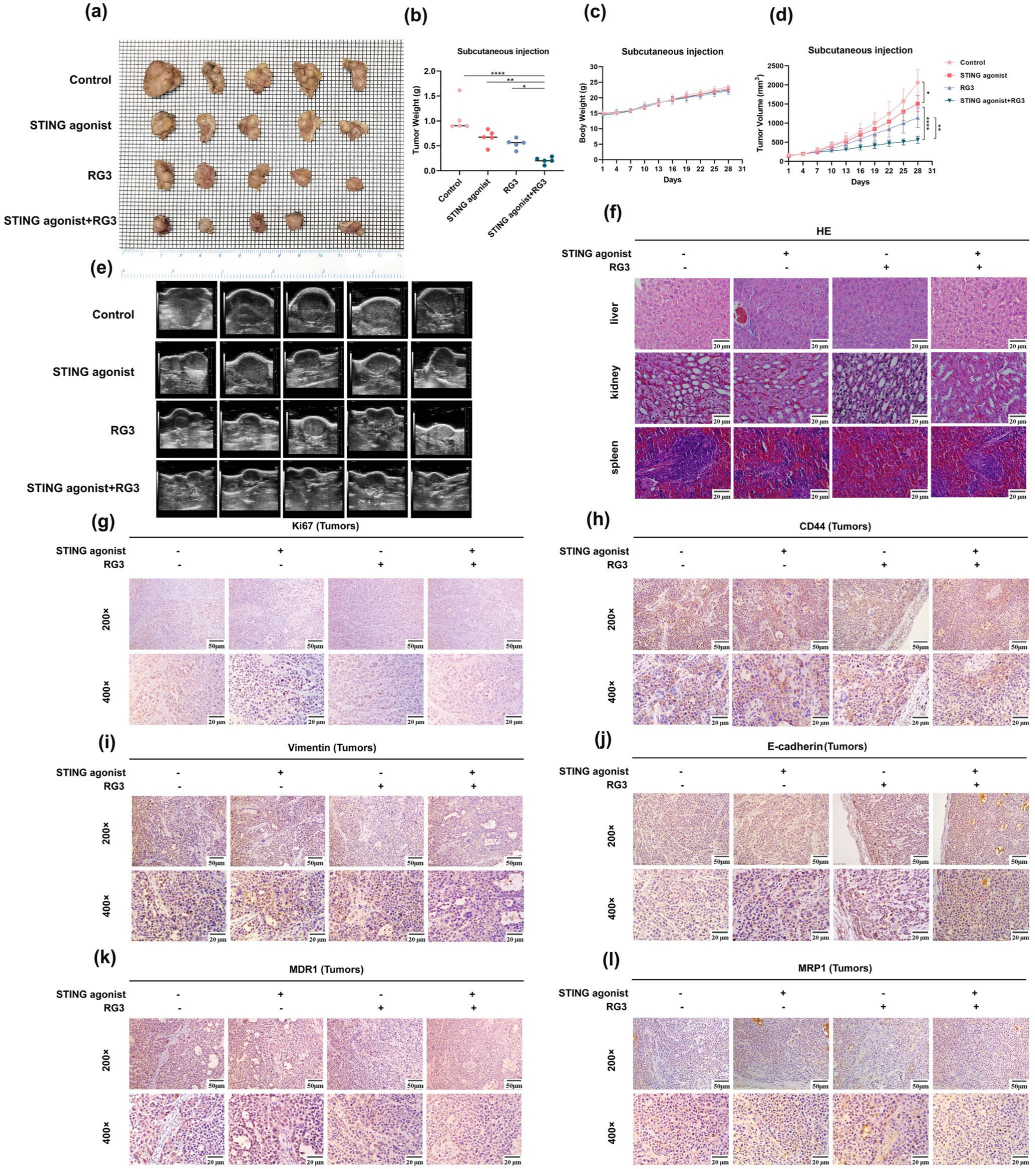
In vivo experiments demonstrating the reversal of cisplatin resistance and inhibition of tumor growth by STING agonist and RG3. (a) Photographic images of tumors in the gastric cancer xenograft model in mice. (b, d) Measurement of tumor weight and volume after subcutaneous injection. (c) Monitoring of mouse body weight after subcutaneous injection. (e) Ultrasound images of mouse tumors after administration. (f) Hematoxylin and eosin (HE) staining of liver, kidney, and spleen tissues after administration. (g–l) Immunohistochemical detection of the expression of Ki67, CD44, Vimentin, E‐cadherin, MDR1, and MRP1 in tumor tissues after subcutaneous injection. (* denotes comparison with the control group. # represents comparison with the STING agonist group. & denotes comparison with the RG3 group. *, #, & indicate statistical significance at *p <* 0.05. **, ##, && indicate statistical significance at *p <* 0.01. ***, ###, &&& indicate statistical significance at *p <* 0.001. ****, #### indicate statistical significance at *p <* 0.0001).

To investigate the effects of STING agonists and RG3 on gastric cancer progression, a xenograft tumor model was established in athymic nude mice using SGC‐7901/DDP cells. The results showed that both the STING agonist c‐di‐AMP and RG3 individually had significant tumor‐suppressive effects relative to the control group. Importantly, the combined treatment with the STING agonist and RG3 exhibited a significantly stronger inhibitory effect on gastric cancer tumors (Figure 6a–e). Once tumor volumes averaged 150–200 mm^3^, tumor‐bearing mice were randomly assigned to four groups: control, STING agonist, RG3, and STING agonist + RG3. Seven treatments were administered every 3 days per group, and ultrasound imaging was performed to monitor tumor progression. Gastric cancer tumor alterations were comprehensively assessed after treatment, including tumor volume, two‐dimensional echogenicity changes, and color Doppler imaging. Tumors in the control group exhibited significant enlargement, indistinct boundaries, irregular morphology, heterogeneous echogenicity, and hypoechoic necrotic areas with angular edges, some extending peripherally. In contrast, tumor growth was notably constrained in the STING agonist, RG3, and combination treatment groups. Tumor growth was most restricted in the combination treatment group, where tumors exhibited well‐defined boundaries, oval shapes, smooth edges, and homogeneous echogenicity. These findings strongly indicate that the combination of STING agonists and ginsenoside RG3 synergistically inhibits gastric cancer progression.

Additional experiments were conducted to investigate the effects of the STING agonist and RG3 on the cisplatin sensitivity of SGC‐7901/DDP cells in the context of tumor growth. A subcutaneous xenograft tumor model was established in mice. Nine male BALB/c nude mice (4–5 weeks old) were randomly assigned to three groups: control, DDP, and STING agonist + RG3 + DDP groups. To establish the subcutaneous xenograft tumor model, 6 × 10^6^ SGC‐7901/DDP cells suspended in Matrigel (BD Biosciences, Franklin Lakes, NJ, USA) and PBS were injected into the right inguinal region of each mouse. Mice were monitored daily for general health, and data on body weight and tumor size were recorded. When the average tumor volume reached 150–200 mm^3^, treatments were administered as follows: the control group received intraperitoneal saline; the DDP group received intraperitoneal cisplatin (5 mg/kg) (Cao et al. 2021; Zhao et al. 2023); and the STING agonist + RG3 + DDP group received intraperitoneal RG3, STING agonist, and cisplatin. Treatments were administered weekly for three injections. Results indicated that cisplatin alone moderately inhibited tumor growth compared to the control group. However, in the combination treatment group, cisplatin resistance in gastric cancer was reversed, significantly inhibiting tumor growth and resulting in gradual tumor shrinkage (Figure S1). In conclusion, the combination of the STING agonist and RG3 effectively reversed cisplatin resistance in gastric cancer, enhanced the cisplatin sensitivity of SGC‐7901/DDP cells during tumor growth, and inhibited tumor progression.

Ultrasound imaging, without the need for labeling, enables precise detection of three‐dimensional structures, radial distances, areas, and tumor volumes as small as 0.1 mm^2^. In tumor research models, it enables real‐time monitoring of tumor size changes alongside vascular distribution, internal echo variations, necrosis, and liquefaction. Additionally, it facilitates the assessment of surrounding tumor tissues and lymph node metastasis. Energy Doppler imaging provides vivid visualization of neovascular growth and distribution, microvessel‐to‐tumor volume ratios, and tumor blood supply, delivering invaluable support for oncological research. Furthermore, in drug administration models, it facilitates real‐time monitoring of drug effects on cardiovascular function in animal models. In this study, ultrasound was periodically utilized to measure mouse tumor size and volume and to assess blood flow distribution. During gastric cancer tumor progression, some control group tumors displayed significant internal echo changes, including non‐uniform hypoechoic areas suggestive of liquefaction or necrosis. Ultrasound scans identified liquefaction and necrosis, potentially correlating with the rapid growth observed in control group tumors. However, the ultrasound imaging equipment used in this study had limitations, failing to adequately capture color blood flow signals after freezing images. This limitation will be systematically addressed in future studies.

Immunohistochemistry was performed to assess the expression of Ki‐67 (proliferation marker), VEGF (angiogenesis marker), E‐cadherin and Vimentin (EMT markers), and CD44 (stemness marker) in mouse tumors. The results were consistent with in vitro findings (Figure 6g–l), confirming that the STING agonist and RG3 combination significantly inhibits gastric cancer progression and reverses drug resistance in vivo. Importantly, no significant differences in body weight or apparent normal tissue damage were detected among the three treatment groups compared to the control group (Figure 6c). HE staining revealed no adverse effects on the organs of mice across experimental groups (Figure 6f). These findings suggest that the STING agonist and RG3 combination is a promising therapeutic strategy for gastric cancer.

## 4 Discussion

Recent clinical trial data underscore the promising efficacy of immunotherapy in gastric cancer, especially with programmed cell death protein 1 (PD‐1) inhibitors (Lordick et al. 2022). Despite these advancements, the evolving therapeutic landscape faces persistent challenges, notably the emergence of primary or acquired resistance, a significant obstacle in the current gastric cancer treatment paradigm. This resistance frequently results in clinical relapse and a poorer prognosis (Xiao et al. 2022). The complexities of multidrug resistance (MDR) in gastric cancer cells involve an intricate interplay of mechanisms, including the dysregulation of apoptosis signaling, loss of cell cycle checkpoint control, accelerated cell proliferation, and altered autophagic flux. Furthermore, enhanced DNA damage repair, reduced drug uptake, and increased drug efflux via the upregulation of MDR‐related proteins contribute to this phenomenon. The activation of cancer stem cells (CSCs) and induction of epithelial‐mesenchymal transition (EMT) are also critical factors in MDR development in gastric cancer (Wei et al. 2020).

The STING signaling pathway is a critical immune signaling cascade essential for immune surveillance, facilitating the detection and response to infections, tumors, and various pathological conditions. Activation of the cGAS‐STING signaling pathway can transform a ‘cold’ tumor microenvironment into a ‘hot’ one by stimulating interferon (IFN) production, offering a promising avenue to enhance immunotherapy against malignant tumors (Guan et al. 2023). A range of synthetically engineered small‐molecule STING agonists has been developed, demonstrating significant amplification potential over inhibitors, which have limitations in suppression. Currently, approximately 10 STING agonists have entered clinical trials, showing significant promise in tumor immunotherapy through activation of the cGAS‐STING signaling pathway (He et al. 2024). Studies suggest that the STING agonist cGAMP not only directly enhances NK cell‐mediated anti‐tumor activity but also increases the sensitivity of pancreatic cancer cells to NK cell cytotoxicity, thereby exerting anti‐tumor effects in this context (Da et al. 2022). Moreover, activation of the cGAS‐STING pathway is associated with increased generation of double‐stranded DNA (dsDNA), reversal of tumor hypoxia, and impairment of DNA damage repair. This increased DNA damage can enhance cancer sensitivity to radiotherapy (Yi et al. 2024). Additionally, STING overexpression has been shown to increase the sensitivity of BRCA‐mutant breast cancer to Poly (ADP‐ribose) polymerase inhibitors (PARPi). Conversely, the downregulation of STING induces resistance to PARPi in BRCA‐mutant breast cancer (Bustos et al. 2023). Inhibition of adenosine‐5′‐triphosphate citrate lyase, leading to peroxidation of polyunsaturated fatty acids (PUFA) and mitochondrial damage, triggers mitochondrial DNA leakage. This, in turn, activates the cGAS‐STING innate immune pathway, overcoming cancer resistance to PD‐L1 therapy in a cGAS‐dependent manner (Xiang et al. 2023). Collectively, these findings highlight the strong association between the cGAS‐STING signaling pathway and both tumor immunotherapy and resistance mechanisms.

Ginsenoside Rg3, a derivative of traditional Chinese medicine, has emerged as a promising herbal compound with notable clinical potential (Li et al. 2022). Rigorously studied, Rg3 has been used in anticancer therapy, demonstrating efficacy across various tumor types, including gastric cancer (Yang et al. 2020), liver cancer (Li et al. 2022), and breast cancer (Zhao, Ma, et al. 2024). Rg3 exerts anticancer effects through diverse mechanisms, including inhibiting tumor cell proliferation, inducing apoptosis, and arresting the cell cycle. Notably, ginsenoside Rg3 enhances tumor cell immunogenicity, promoting differentiation and effectively inhibiting malignant metastasis and invasion (Im 2020). Given the strong link between angiogenesis and tumor progression, Rg3 is effective in hindering the advancement of precancerous lesions in gastric cancer by suppressing angiogenesis (Zeng et al. 2022). This multifaceted impact makes ginsenoside Rg3 a valuable therapeutic agent in cancer treatment. Studies show that Rg3 plays a key role in reducing multidrug resistance in tumor cells and enhancing their sensitivity to anticancer drugs through various mechanisms, including pathway activation and synergistic interactions with other drugs (Chen et al. 2022; Ni et al. 2022). After extensive preclinical studies, ginsenoside Rg3 has moved into clinical treatments for cancer patients in China, showing promising prospects. Its use, particularly as part of the Shenqi Fuzheng Injection combined with chemotherapy, has proven effective in treating digestive system cancers. Results show that this combination therapy enhances clinical efficacy and mitigates chemotherapy‐induced side effects in these cancers (Pan et al. 2019). Additionally, in patients with advanced hepatocellular carcinoma (HCC) and adequate liver function, combining transcatheter arterial chemoembolization (TACE) with ginsenoside Rg3 has shown potential to significantly prolong overall survival compared to TACE alone (Zhou et al. 2016). These clinical studies collectively highlight the robust anticancer properties of Rg3, making it a valuable component in comprehensive cancer treatment strategies.

In our previous studies, we explored the roles of the cGAS‐STING signaling pathway and ginsenoside Rg3 in cancer therapy, highlighting their potential to enhance tumor immunotherapy and exhibit significant anticancer effects (Lu et al. 2022; Chang et al. 2023). Building on this foundation, our current research focuses on combining the STING agonist c‐di‐AMP with ginsenoside Rg3 to evaluate their combined impact on cisplatin‐resistant gastric cancer cells (SGC‐7901/DDP). Through a series of in vitro and in vivo experiments, we evaluated various aspects of cancer progression, including cell proliferation, migration, angiogenesis, apoptosis, stemness, epithelial‐mesenchymal transition (EMT), and drug resistance. Our findings showed that the combination of the STING agonist c‐di‐AMP and ginsenoside Rg3 synergistically inhibited cell proliferation and migration, suppressed angiogenesis, hindered stemness and EMT, and promoted apoptosis in SGC‐7901/DDP cells. Furthermore, this combination effectively reversed cisplatin resistance in the cells. In summary, our study highlights the potential of the STING agonist c‐di‐AMP, in synergy with ginsenoside Rg3, to impede the progression of SGC‐7901/DDP cells, providing a promising avenue for future cancer therapeutics. Despite the therapeutic potential of Rg3 and STING agonists, challenges exist due to Rg3's limited water solubility, poor oral bioavailability, and STING‐related tumor immune tolerance (Wan et al. 2023). Recent advancements in drug delivery systems, particularly nano‐delivery systems, offer promising solutions to enhance drug efficacy and reduce toxicity (El‐Banna et al. 2022). Building on the insights from our research, our group plans to develop a nano‐platform that can carry the STING agonist c‐di‐AMP and the traditional Chinese medicine ginsenoside Rg3. This innovative approach aims to overcome the limitations of their administration, focusing on improving anti‐tumor effects and addressing issues related to tumor drug resistance. Subsequent studies will further explore the potential of this nano‐platform in cancer therapy.

## 5 Conclusion

In conclusion, our study highlights the synergistic potential of the STING agonist c‐di‐AMP in combination with the traditional Chinese medicine ginsenoside Rg3 to effectively reverse cisplatin resistance in SGC‐7901/DDP cells. This collaborative approach presents a novel and promising strategy for immunotherapy, particularly in addressing the clinical challenges posed by chemotherapy‐resistant gastric cancer. Further exploration and validation of this combination could pave the way for innovative and more effective therapeutic interventions in the treatment of gastric cancer.

## Author Contributions

**Zhongqi Lu:** conceptualization (equal), data curation (equal), formal analysis (equal), methodology (equal), resources (equal), software (equal), supervision (equal), writing – original draft (equal). **Yihang Fu:** data curation (equal), software (equal), validation (equal). **Qiang Fu:** conceptualization (equal), data curation (equal), formal analysis (equal), investigation (equal), resources (equal), software (equal). **Ying Chang:** conceptualization (equal), formal analysis (equal), validation (equal). **Tiefeng Jin:** conceptualization (equal), funding acquisition (equal), writing – review and editing (equal). **Meihua Zhang:** funding acquisition (equal), resources (equal), visualization (equal), writing – review and editing (equal).

## Ethics Statement

All applicable international, national, and/or institutional guidelines for the care and use of animals were followed. All experimental procedures and animal care have been approved by the Laboratory Animal Ethics Committee Yanbian University and conducted in accordance with the guidelines outlined by the National Institutes of Health on the care and use of laboratory animals (The IACUC Issue No.is YD20240125001).

## Conflicts of Interest

The authors declare no conflicts of interest.

## Supporting information


**Figure S1.** The combination of the STING agonist and RG3 effectively reversed cisplatin resistance in gastric cancer and increased the sensitivity of SGC‐7901/DDP cells to cisplatin during tumor growth, thereby inhibiting tumor progression. (a and b) Gross examination of tumor‐bearing mice and gross appearance of tumor tissues. (c) Weight statistics of tumors in each group. (* indicate statistical significance at *p <* 0.05. ** indicate statistical significance at *p <* 0.01. *** indicate statistical significance at *p <* 0.001).

## Data Availability

The data underpinning the findings of this study can be obtained from the corresponding author upon request.
