# Supplementary figures and images for: Ginsenoside RG3 Synergizes With STING Agonist to Reverse Cisplatin Resistance in Gastric Cancer
Source: Food Sci Nutr. 2025 Jan 20;13(1):e4744. doi: 10.1002/fsn3.4744 (PMC11745231; doi:10.1002/fsn3.4744)

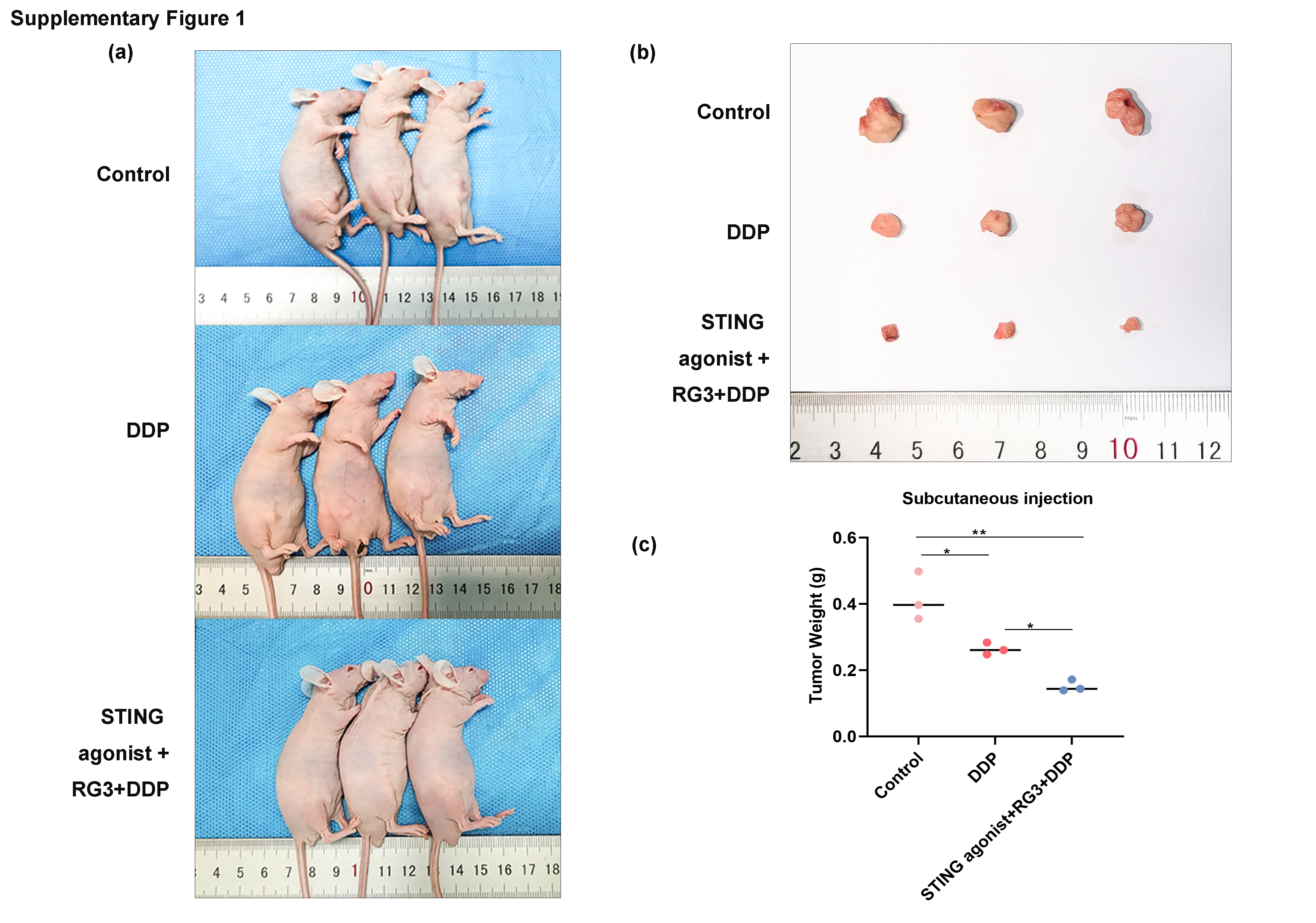

Supplement: Supplementary file 1 — Figure S1. The combination of the STING agonist and RG3 effectively reversed cisplatin resistance in gastric cancer and increased the sensitivity of SGC‐7901/DDP cells to cisplatin during tumor growth, thereby inhibiting tumor progression. (a and b) Gross examination of tumor‐bearing mice and gross appearance of tumor tissues. (c) Weight statistics of tumors in each group. (* indicate statistical significance at p < 0.05. ** indicate statistical significance at p < 0.01. *** indicate statistical significance at p < 0.001). [file FSN3-13-e4744-s001.jpg]
